# A Comparison of Frequency- and Agreement-Based Response Formats in the Measurement of Burnout and Engagement

**DOI:** 10.3390/ijerph17020543

**Published:** 2020-01-15

**Authors:** Jiajin Tong, Robert M. Bickmeier, Steven G. Rogelberg

**Affiliations:** 1 School of Psychological and Cognitive Sciences, Beijing Key Laboratory of Behavior and Mental Health, Peking University, Beijing 100871, China; 2 Organizational Science, University of North Carolina at Charlotte, Charlotte, NC 28223, USA

**Keywords:** burnout, engagement, frequency response format, agreement response format, usefulness analysis, relative importance analysis

## Abstract

The present research compares and contrasts frequency versus agreement response formats, two approaches to measuring job burnout and work engagement. Construct-based and measurement-based arguments for the superiority of the frequency response format in measuring burnout/engagement are provided, demonstrating that frequency-based measurements will explain relatively more variance in outcome variables. Fair comparison, time order counterbalance, and multiple measuring waves justify the comparison and reduce common method errors of self-report measures. Sample 1 (*N* = 242) was composed of employees from multiple organizations, while the participants in Sample 2 (*N* = 281) were employees from one company. Relative importance analysis showed that frequency outperforms the agreement response format in measuring burnout and engagement in both samples. These findings suggest that the frequency response format provides a more valuable method of detecting the dynamic nature of burnout/engagement, which offers methodological guidance for future research involving dynamic constructs. These findings can lead to improvements in the measurement of the dynamic experiences of burnout and engagement. This is one of the first studies to provide evidence whether the dynamic nature of the constructs would have any bearing on the response formats.

## 1. Introduction

Job burnout and work engagement are both intense personal experiences and research topics in occupational, organizational, and health psychology, affecting a variety of populations such as workers [1], social workers [2], and students [3]. Both variables consist of complex, dynamic states, and our current approach to measurement may not adequately adapt to the dynamic components of burnout symptoms and engagement experiences. Thus, we propose a simple alternative to measuring burnout and engagement: Frequency-based response scales. In the following studies, we test and provide evidence suggesting that frequency-based response scales (e.g., those designed to indicate how often a target behavior or symptom occurs) rather than agreement-based response scales (e.g., those designed to assess how intensely the respondent agrees that a given symptom or experience has occurred) are better-suited for assessing the dynamic elements of burnout and engagement. Consequently, we can achieve a richer understanding of how burnout and engagement are experienced, and how they relate to variables of interest, by improving our approach to measurement.

### 1.1. Measuring Dynamic Constructs

Variables such as affect and behavior are usually measured by a unipolar scale of frequency, ranging from “Never” to “Always” in the extant literature (e.g., UWES [4]), while trait variables such as beliefs, values, and personality are usually measured by a bipolar scale of agreement, ranging from “strongly negative” to “strongly positive” (e.g., Big Five [5]). Agreement scales lend themselves to trait measurement because of the relatively high stability of traits. States, however, can be momentary and variable [6]. Fluctuations can occur from day to day or even moment to moment (e.g., [7,8]).

Burnout was first defined as a stable syndrome [9], yet recent research has shown that both burnout and engagement demonstrate dynamic qualities (e.g., [7,8]). For example, some scholars proposed dynamic components (i.e., task-level view of engagement) out of the general construct (job-level view) and observed that task-level engagement can “spill-over” to subsequent tasks within a job [10]. In addition, the dialectical perspective on burnout and engagement argues that burnout and engagement can occur simultaneously and independently within-person as separate, dynamic states [11]. As such, appropriate measurement tools are needed to assess the fluctuating nature of burnout/engagement. Either the agreement (i.e., a bipolar scale, ranging from “strongly disagree” to “strongly agree”) or frequency response format (i.e., a unipolar scale, ranging from “never” to “daily/always”) may be employed in measuring dynamic psychological states (e.g., [12]). It is unclear, however, whether agreement or frequency response formats are superior approaches to capturing dynamic psychological states. Because different response format may lead to different scores for the same measuring item, it is critical for researchers to identify the response format best-suited for their instruments and corresponding research questions.

Past research has compared rating scale performance in educational (e.g., [13]) and health-function testing (e.g., [12]). But all previous research has been cross-sectional and focused on the psychometrics of item measurement, based on item response theory, rather than comparing the validity of different rating responses. No research yet focuses on the response format differences of burnout and engagement. Moreover, past research has not considered whether the nature of the constructs (e.g., dynamic states) would have any bearing on whether the frequency or agreement response format better fits the construct. Identifying the best-fit set of response labels and item design should facilitate greater accuracy in comparisons within and between individuals and across studies. Response options that encourage inference and estimation strategies may interfere with such comparisons and encourage judgments that do not accurately represent the respondent’s daily life [14]. Thus, we compare the performance of agreement response scales and frequency response scales (vague or precise) in the ensuing studies to provide data in support of identifying the response format best-suited for burnout and engagement research.

### 1.2. Frequency vs. Agreement Scales

Brown [13] notes that participants respond to both frequency and agreement response formats by recalling (ideally) relevant information from memory. With dynamic or fluctuating phenomena, respondents may need to average the fluctuating levels of the construct of interest, and the respondents’ averaging strategy differs between frequency and agreement formats. Once the respondents have successfully calculated an average, they must then identify the appropriate, fitting response from the options available. Agreement formats (e.g., slightly agree, agree, strongly agree) can be vague and highly subjective, leading participants to rely on varying strategies to calculate fluctuations in the relevant phenomena [13]. For example, respondents may rely on reporting the number of times a particular phenomenon occurred, or they may rely on the degree of intensity in which they experienced the phenomenon. In other words, a respondent may select “agree” to indicate that they “feel somewhat drained from work” several times per week, while another respondent may also select “agree” to indicate they “feel somewhat drained from work” intensely, yet infrequently or even only once per week. Naturally, these differing response strategies create challenges in interpreting measurements when using agreement scales.

In fact, the response labels may more strongly influence the respondents’ calculations to the extent that instances are more difficult to recall, for example, because of a long recall period [14]. Put differently, respondents are more likely to use the response labels as a source of information to determine the appropriate response to the item (instead of the actual frequency of the behavior targeted by the item) when recall of such instances may be difficult. In summary, agreement response scales may be vague and promote simple average and estimate strategies over specific calculations so that respondents can more easily and efficiently respond to survey items [14,15,16]. These concerns suggest that frequency responses may be better-suited to measuring the dynamic elements of burnout and engagement than agreement responses. Scholars also suggest that frequency-type ratings can adequately reflect respondents’ general feelings with respect to burnout and engagement [17]. Thus, we propose the following hypotheses:

**Hypothesis** **1.** *Frequency-based measurements of burnout/engagement will explain significant, incremental variance beyond agreement-based measure of burnout/engagement in the selected outcome variables.*

**Hypothesis** **2.** *Frequency-based measurements of burnout/engagement will account for a significantly greater proportion of the variance (i.e., relative importance) than agreement-based measure of burnout/engagement in the selected outcome variables.*

### 1.3. Analytical Strategy for Gauging Relative Importance

Traditional measures of relative importance (e.g., simple correlation comparison—*r*, squared standardized regression weights—β^2^) may fail to adequately characterize the relative contribution/importance of predictors when the variables are highly inter-correlated (i.e., two groups of predictors in this case [18]). When comparing the relative contributions of two measures, the practical questions are: (1) Does either measure have unique variance in the criterion variable above and beyond that of the other measure in the regression model? (2) What is the contribution of each measure in the presence of the other? Thus, we examine both incremental validity and relative importance in the context of our research foci [19].

On its own, analyzing incremental validity attributes any shared criterion-related validity to the earlier-step measure and none to the later-step measure [19]. Relative importance analysis can complement incremental validity analysis because relative importance analysis provides estimates of importance scaled in the metric of relative effect sizes (i.e., proportion of predictable criterion variance attributed to each predictor/measure) without relying on sample-size-dependent significance tests. Consequently, including information both about a predictor’s incremental importance and relative importance will permit people to evaluate the statistical quality of the predictors and to illustrate the overall contribution of the predictor of interest in a more balanced manner [19].

Relative weights rather than general dominance weights will be used to calculate relative importance in this study, because the former offers an advantage when a large number of predictors are involved (e.g., more than 10 predictors in the regression model [19]).

## 2. Methods

### 2.1. Participants

We sampled employees from organizations in the service industries (i.e., government or government financed institutions, schools) or in business companies (i.e., cross-national companies). For the following study, we recruited two samples. For Sample 1, we requested contact persons to recruit participants randomly chosen from their respective organizations. For Sample 2, we requested our contact person to recruit participants from stores randomly chosen from their organization.

Sample 1 was composed of 242 employees from different organizations in China. Participants ranged from 20 to 53 years old, with a mean age of 32.1 (SD = 5.9) years. In this sample, 33% of participants were male. All participants were general employees (without any management titles). Participants’ tenure ranged from 0.1 to 33 years, with a mean of 9.5 (SD = 6.6) years.

Sample 2 was composed of 281 employees from seven stores of a cross-regional company in China. Participants ranged from 18–44 years old, with a mean age of 25.35 (SD = 4.75) years. A total of 145 participants (52%) were male; 180 participants (64%) had less than an undergraduate education. Participants’ tenure ranged from 1 to 348 months, with a mean of 49.12 (SD = 51.26) months.

### 2.2. Procedure

This research compared the predictive and explanatory power of agreement response formats, vague frequency response formats, and precise frequency response formats of burnout/engagement measures with respect to empirical criteria from both work- and family-domains (e.g., job satisfaction, sleep quality). Sample 1 participants were requested to rate their burnout/engagement in “the last two weeks”, while Sample 2 participants were asked to consider “the past in general”. The levels of difficulty are different in averaging the dynamic states of burnout/engagement in these two cases. Each sample provided data from three time points (separated by two-week intervals).

In both samples, we used response formats with identical score ranges (distributional equivalence) across the exact same items for the same factor structure and same constructs (procedural equivalence [20]). In addition, we counterbalanced the presentation of each response format. We asked each participant to rate their respective items using the frequency format at one time and the agreement format at another time, to avoid any confusion that might arise by asking participants to provide two sets of ratings on the same items simultaneously. Ideally, our counterbalanced design should minimize any common method variance (e.g., [21]) or spillover effects.

Participants were randomly assigned to two groups (n_1_ = 122, n_2_ = 120 in Sample 1 and n_1_ = 145, n_2_ = 136 in Sample 2) for counterbalancing. Results showed that the two groups did not have significant differences in any of the burnout or engagement dimensions in either sample (all *p*s < 0.05). Participants in Group 1 used the unipolar frequency response at Time 1 and bipolar agreement response at Time 2 (two weeks later), while participants in Group 2 used the bipolar agreement response at Time 1 and unipolar frequency response at Time 2 to rate burnout/engagement. Another two weeks later, at Time 3, all participants completed scales on outcome variables (e.g., CWB, OCB). The specific measures were administered as indicated in Table 1.

Participants completed their surveys anonymously on-line with substitute random code to indicate each participant for different waves. In Sample 1, our contacts sent out a total of 309 questionnaires and 242 (Group 1 = 122 and Group 2 = 120) completed and valid questionnaires were returned, (response rate of 78% for Time 1); 242 were sent out and 219 (Group 1 = 108 and Group 2 = 111) were returned for Time 2 (91% response rate), and 219 were sent out with 209 (Group 1 = 105 and Group 2 = 104) returned for Time 3 (95% response rate). In Sample 2, the response rates were 81% (349 sent out with 281 valid returned, Group 1 = 145 and Group 2 = 136), 77% (281 sent out with 216 returned, Group 1 = 122 and Group 2 = 94), and 87% (216 sent out with 188 returned, Group 1 = 98 and Group 2 = 90) for respective survey waves. No cases were discarded due to missing data.

### 2.3. Measures

Job Burnout Survey I (unipolar frequency response format). We selected the Maslach Burnout Inventory-General Survey (MBI-GS [22]) to measure two core factors of job burnout: exhaustion (α = 0.91 and 0.88 in Sample 1 and 2) and cynicism (α = 0.86 and 0.87 in Sample 1 and 2). We omitted the inefficacy dimension because the research is unclear regarding its overall fit with the burnout construct (e.g., [23,24]) when measured by the Maslach Burnout Inventory-General Survey (MBI-GS [22]) and the Utrecht Work Engagement Scale (UWES [4]). We asked participants to rate the frequency of the listed feelings and behaviors on a frequency scale with relatively vague quantifiers (1 = never, 2 = almost never, 3 = rarely, 4 = moderately/sometimes, 5 = frequently, 6 = very frequently, 7 = always/every day) in Sample 1 and with more precise quantifiers (1 = never, 2 = a few times a year or less, 3 = once a month or less, 4 = a few times a month, 5 = once a week, 6 = a few times a week, 7 = everyday) in Sample 2. Example items include “I feel drained from my work” and “I doubt the significance of my work”.

Work Engagement Survey I (unipolar frequency response format). We assessed work engagement using the Utrecht Work Engagement Scale (UWES [4]). We selected the core dimensions of vigor (α = 0.85 and 0.91 in Sample 1 and 2) and dedication (α = 0.89 and 0.88 in respective samples), because they correspond to the dimensions assessed with the selected burnout measure (e.g., [23,24]). We repeated the instructions of the MBI-GS for the participants as they completed the UWES. Example items include “At my job, I feel strong and vigorous” and “I find the work that I do full of meaning and purpose”.

Job Burnout Survey II (unipolar frequency response format). We chose the Shirom–Melamed Burnout Measure (SMBM [25]) to measure job burnout in Sample 2. The SMBM measured the following dimensions: Physical Fatigue (α = 0.94), Cognitive Weariness (α = 0.95), and Emotional Exhaustion (α = 0.91). We asked participants to follow the same instructions as the MBI-GS for Sample 2. Example items include “I feel physically drained”, “My thinking process is slow”, and “I feel I am unable to be sensitive to the needs of coworkers and customers”.

Work Engagement Survey II (unipolar frequency response format). We used the Shirom–Melamed Vigor Measure (SMVM [26]) to measure work engagement. The SMVM included the following dimensions Physical Strength (α = 0.96), Cognitive Liveliness (α = 0.92), and Emotional Energy (α = 0.92). Again, participants followed the same instructions as the MBI-GS for Sample 2. Example items include “Feeling vigorous”, “I feel I can think rapidly”, and “I feel able to be sensitive to the needs of coworkers and customers”.

Work Engagement and Job Burnout Survey I and II (bipolar agreement response format). Using the same inventory items as the unipolar frequency response format selections, (exhaustion, α = 0.89 and 0.85; cynicism, α = 0.82 and 0.85; vigor, α = 0.80 and 0.82; dedication, α = 0.88 and 0.85, in respective samples; and physical fatigue, α = 0.93, cognitive weariness, α = 0.94; emotional exhaustion, α = 0.91; physical strength, α = 0.91; cognitive liveliness, α = 0.88; emotional energy, α = 0.92, in Sample 2), participants rated individual agreement to the listed feelings and behaviors on an agreement scale (1 = strongly disagree, 4 = neutral, 7 = strongly agree).

### 2.4. Control and Criterion Variables

We controlled for demographic variables (e.g., age, gender, tenure, education level, which were found to be potentially related to burnout/engagement [9]) in both samples. In sample 2, we additionally controlled for body mass index (BMI) because BMI relates to health functioning and sleep quality/disorder (e.g., [27]).

Our criterion variables include behaviors, attitudes, and results, including both work- and family-domain outcomes. Research has previously established a relationship between our chosen criterion variables and burnout/engagement (e.g., job satisfaction and organizational commitment [28,29,30]; contextual performance/organizational citizenship behavior [29,31]; incivility/counterproductive work behavior [32]; sleep quality/disorder and work-family conflict [33]). Because work-family positive spill-over is the opposite of work-family conflict, and it is related to mental health [34], it should also be related to burnout/engagement. Participants were requested to rate CWB, OCB, Sleep Quality/Disorder, Work-family Positive Spillover and WFC items on a frequency scale (following the same quantifiers in MBI-GS in respective samples) and to indicate their General Job Satisfaction and Organizational Affective Commitment in an agreement scale (1 = strongly dissatisfied/disagree to 7 = strongly satisfied/agree).

Counterproductive Work Behavior. We measured counterproductive work behavior (CWB) using Fox and Spector’s [35] CWB measure. Example items include “Tried to look busy while doing nothing” (from Organizational Deviance, α = 0.61 and 0.81 in Sample 1 and 2) and “Insulted someone about their job performance” (from Interpersonal Deviance, α = 0.71 and 0.96 in Sample 1 and 2).

Organizational Citizenship Behavior. We adapted an 8-item measure of organizational citizenship behavior (OCB, α = 0.92 and 0.83 in Sample 1 and 2) from the organizational citizenship behavior scales of Smith, Organ, and Near [36] (i.e., “Helps other employees with their work when they have been absent”, “Makes innovative suggestions to improve the overall quality of the department”, “Assists the supervisor with his/her duties”), from Podsakoff, Ahearne, and MacKenzie [37] (i.e., “Willingly share their expertise with colleagues”; “Willingly give of their time to help colleagues who have work-related problems”; “Encourage colleagues and give them positive feedback”; “Pay attention when colleagues describe work-related problems”), and from Farh, Zhong, and Organ [38] (i.e., “Willing to coordinate and communicate with colleagues”).

General Job Satisfaction. We used Evers, Frese, and Cooper’s [39] 10-item scale (α = 0.89 and 0.82 in Sample 1 and 2) to measure general job satisfaction. Example item includes: “The style of supervision.”

Organizational Affective Commitment. We assessed organizational affective commitment (α = 0.87 and 0.92 in Sample 1 and 2) with Chen and Francesco’s [40] six-item measure. An example item was “I really feel as if this organization’s problems are my own.”

Sleep. We followed the recommendations of the Diagnostic and Statistical Manual of Mental Disorders [41] to measure sleep quality (α = 0.67 in Sample 1). We adapted items from the Pittsburgh Sleep Q uality Index [42]. The items were “I easily go to sleep at night”, “I wake up naturally in the morning”, and “I have good sleep quality at night”. We measured symptoms of sleep disorder (α = 0.93 in Sample 2) with seven items from the Karolinska Sleep Questionnaire [43]. Sample items include “difficulties falling asleep” and “not well-rested on awakening.”

Work-family Positive Spillover. We measured work-family positive spillover (α = 0.96 in Sample 2) with six items developed by Hanson, Hammer, and Colton [34]. Sample items include “Abilities developed at work help me in my family life.”

Work-family Conflict. We measured work-family conflict (WFC, α = 0.87 in Sample 2) with five items developed by Carlson, Kacmar and Williams [44]. Sample items include “My work keeps me from my family activities more than I would like.”

## 3. Results

### 3.1. Confirmatory Factor Analysis

Results of confirmatory factor analysis showed that the eight-factor measurement model (i.e., frequency Exhaustion, Cynicism, Vigor, and Dedication, and agreement counterparts) fits the data adequately in Sample 1 (*x*^2^ = 513.83, *df* = 224, *CFI* = 0.93, *TLI* = 0.92, *RMSEA* = 0.07, *SRMR* = 0.06) and the twenty-factor measurement model (i.e., frequency Exhaustion, Cynicism, Vigor, and Dedication, and frequency Physical Fatigue/Strength, Cognitive Weariness/Liveliness, Emotional Exhaustion/Energy, and all the agreement counterparts) fits the data adequately in Sample 2 (*x*^2^ = 2948.85, *df* = 1520, *CFI* = 0.91, *TLI* = 0.90, *RMSEA* = 0.06, *SRMR* = 0.04).

### 3.2. Preparing for Hypothesized Model Analysis

Means, standard deviations and correlations are provided in Table 2 and Table 3 for respective samples. Relative weights and incremental importance statistics are listed in Table 4 and Table 5, providing comparisons with cluster variables listed as “total-agreement” and “total-frequency”. The integrated tables with detailed variable level statistics information on multiple regressions (e.g., *r* and *β*) and relative importance (e.g., rescaled estimates, individual incremental importance) can be provided on request. We omitted comparisons for those models with insignificant squared semi-partial correlation (i.e., Δ*R*^2^), when including burnout/engagement variables (e.g., the models predicting Interpersonal Deviance in Sample 1 and predicting General Job Satisfaction in Sample 2). For the other significant models, usefulness analysis showed that after controlling for demographic variables (i.e., age, gender, tenure) in Sample 1, frequency format burnout/engagement variables explained unique variance beyond agreement format predictors in four out of five significant models (i.e., Organizational Deviance, OCB, General Job Satisfaction, Organizational Affective Commitment), whereas with the agreement format the incremental importance was only significant in two out of five models (i.e., General Job Satisfaction, Organizational Affective Commitment).

After controlling for demographic variables (i.e., age, gender, tenure, education level) and BMI in Sample 2, frequency predictors explained more than agreement variables in all eight relationships for Survey I and all five relationships for significant models measuring with Survey II (i.e., Organizational and Interpersonal Deviance, OCB, Organizational Affective Commitment, Sleep Disorder), whereas agreement variables explained beyond frequency predictors in none of the eight relationships for Survey I and only one out of five significant relationships for Survey II (i.e., Sleep Disorder). Thus, burnout/engagement measured with frequency response explains unique variance in related outcomes beyond agreement response (especially in Sample 2).

### 3.3. Relative Importance Analysis

Relative importance analysis for the five significant models in Sample 1 showed that the frequency format of burnout/engagement measures explained greater variance in organizational deviance (*R*^2^ = 0.06, 80%), OCB (*R*^2^ = 0.10, 78%) and sleep quality (*R*^2^ = 0.05, 58%) than the agreement format. Compared to agreement responses, frequency responses explained similar amounts of variance in general job satisfaction (*R*^2^ = 0.15, 53%) and organizational affective commitment (*R*^2^ = 0.17, 52%). Although the agreement format explained more in interpersonal deviance, the total explained variance was only marginally significant. Results of this model were therefore omitted in the consideration of measurement comparison.

In Sample 2, relative importance analysis showed that frequency-based measurements of burnout/engagement outperform agreement counterparts in all eight criteria for Survey I (*R*^2^ ranging from 0.05–0.12), with the importance percentage ranging from 76–89% (shown in Table 4. Frequency responses outperform agreement responses in seven out of eight criteria for Survey II (*R*^2^ ranging from 0.05–0.18), with the importance percentage ranging from 60–78% (except for Sleep Disorder: when predicting Sleep Disorder, both formats explained similar amounts of variance, see Table 5).

The above results showed that burnout/engagement measured with frequency response did explain more variance or at least as much variance in related outcomes when compared with agreement response, including both unique (incremental variance) and combined variance (relative importance variance) in the presence of agreement response predictors.

## 4. Discussion

### 4.1. Main Findings and Implications

Previous research used either agreement or frequency response format in burnout/engagement research. This research compares frequency and agreement response formats in measuring burnout and engagement, covering both organization- (e.g., OCB, CWB) and family-domain criteria (e.g., WFC). Through incremental and relative importance analysis [19], we observed that frequency-based measurements of burnout/engagement outperformed agreement counterparts in predicting most criteria (with significant models) and was at least as valuable in predicting other criteria (e.g., General Job Satisfaction, Organizational Affective Commitment in Sample 1 and Sleep Disorder in Sample 2). Both Hypothesis 1 and 2 were basically supported. According to the extant research, participants responding to the agreement response formats likely rely on varying strategies to calculate fluctuations in the relevant phenomena [13], especially for the long recall period [14], which leads to less variance captured in the outcome.

However, we observed that frequency-based measurements had similar predictive power to the agreement-based measurements for some of our criteria. Those criteria mostly consisted of general attitudes and biological responses, both of which may be influenced by both dynamic, momentary changes and longer-term, underlying conditions. In accordance with previous research [6,7,8], we suggest that frequency-based measurements would be better-suited for explaining the variance of dynamic statuses (e.g., OCB, CWB, WFC), rather than good at explaining the variance of those general attitudes and long-term conditions. Our findings are similar to previous literature observing that domain-specific measures are not more important than general measures in predicting general job satisfaction and organizational affective commitment [45].

In our study, frequency-based measurements of burnout/engagement outperform agreement counterparts in predicting both frequency- (e.g., OCB) and agreement responses (e.g., Organizational Affective Commitment in Sample 2) among criterion variables. In addition, the precise indicators used in Sample 2 (e.g., “once a week”) appeared to outperform the vague indicators in Sample 1 (e.g., “sometimes”, “frequently”), suggesting that precise frequency indicators may be a better option to measure burnout and engagement and to explain variance in criterion variables. These findings are consistent with past research (e.g., [13,46]) and our own predictions.

This study advances research in rating scale performance comparison (e.g., [7,8]). To date, the previous literature regarding scale comparisons emphasized understanding the underlying processes which determine how individuals respond to an item based on its scale. Our paper builds on that literature to demonstrate the practical differences between frequency- and agreement-based response scales while respecting both distributional and procedural equivalence [20]. Specifically, this research adopted a fair comparison method to draw comparisons between frequency- and agreement-based measurements of burnout/engagement, counterbalanced the measurement orders of comparative scales to reduce time frame variation, separated the measures into different waves to reduce possible common method errors, and measured both burnout/engagement and extended outcomes to indicate the effectiveness of measurements. We further advance the research by identifying a set of conditions in which we expect to observe the greatest performance difference between the response types: When our measurement needs require us to assess burnout and engagement symptoms and experiences over time (i.e., during the past two weeks for a respondent).

Practically, this work will help people find an appropriate and reliable way to measure the dynamic elements of burnout/engagement and to detect individual psychological health functioning. That means we can expand the scope of our burnout and engagement assessments from the mere presence of symptoms to clarify the frequency, duration, and change in symptoms. These data can enable practitioners to identify precipitating burnout events and the effectiveness (both immediately and potentially long-term) of interventions. It may help us distinguish the contribution of severity versus frequency or duration of symptoms in the overall experience of burnout and engagement. In other words, our paper provides insights to burnout and engagement measurement that can inform assessment and intervention design.

### 4.2. Limitations and Future Research

Burnout and engagement exhibit both dynamic and fluctuating experiences and symptoms, making the measurement of such variables easily confounded by time. Sample 1 participants completed their measures in a specific time frame reference (i.e., the past two weeks), and Sample 2 participants were asked to complete their measures with respect to the past in general. In our study, frequency-based scales outperformed agreement scales by a greater margin in Sample 2 than in Sample 1. We cannot parse out the potentially confounding effects of time frame with our current design, though we argue that frequency scales may be better suited to measuring dynamic variables like engagement and burnout, especially as the time frame for participant recall increases. Respondents may more heavily rely on averaging strategies when responding with agreement scales, thereby confounding measurement (e.g., infrequent, severe symptoms may be scored the same as frequent, mild symptoms by a given respondent). The concept of time (such as rating window and duration of recall) should be taken into consideration in the new approach of burnout and engagement research [17].

Future research measuring burnout/engagement needs to take response formats into consideration because of the dynamic nature of the constructs and the cognitive processes undertaken by respondents. Specifically, future research should address the effects of time frame and overall length of recall (e.g., comparing specific time frames to the past in general).

## 5. Conclusions

The present research offers insight into the performance of response formats of measures of burnout/engagement at work. Our data suggest that the format we choose for response options can affect the inferences we draw regarding our variables of interest. With respect to burnout and engagement, we conclude that frequency-based response scales are better suited for measurement of the dynamic aspects of these constructs and explaining the variance associated with their covariates, which opens new doors for burnout and engagement research.

## Figures and Tables

**Table 1:** Measures delivered in different waves among two samples.

Sample	Group	*N*	Wave 1	Wave 2	Wave 3
1	1	122	Unipolar frequency: MBI-GS, UWES	Bipolar agreement: MBI-GS, UWES	Outcomes: CWB(f), OCB(f), JS(a), OC(a), SQ(f)
	2	120	Bipolar agreement: MBI-GS, UWES	Unipolar frequency: MBI-GS, UWES	Outcomes: CWB(f), OCB(f), JS(a), OC(a), SQ(f)
2	1	145	Unipolar frequency: MBI-GS, UWES, SMBM, SMVM	Bipolar agreement: MBI-GS, UWES, SMBM, SMVM	Outcomes: CWB(f), OCB(f), JS(a), OC(a), SD(f), WFS(f), WFC(f)
	2	136	Bipolar agreement rating: MBI-GS, UWES, SMBM, SMVM	Unipolar frequency rating: MBI-GS, UWES, SMBM, SMVM	Outcomes: CWB(f), OCB(f), JS(a), OC(a), SD(f), WFS(f), WFC(f)

Note. JS = job satisfaction; OC = organizational affective commitment; SQ = sleep quality; SD = sleep disorder; WFS = work-family positive spillover; WFC = work-family conflict. Other abbreviations were shown in the text. “f” in the brackets indicates frequency response, while “a” indicates agreement response. Participants were requested to rate items “in the last two weeks” in Sample 1 while “in the past in general” in Sample 2.

**Table 2:** Mean, standard deviation, and correlation matrix for Sample 1 (*N* = 242).

Variable	M (SD)	1	2	3	4	5	6	7	8	9	10	11	12	13	14
1. Exhaustion(f)	3.31 (1.29)	233													
2. Cynicism(f)	3.18 (1.16)	0.65 **	233												
3. Vigor(f)	3.73 (1.07)	−0.27 **	−0.33 **	233											
4. Dedication(f)	3.79 (1.20)	−0.33 **	−0.49 **	0.81 **	233										
5. Exhaustion(a)	3.65 (1.30)	0.67 **	0.52 **	−0.23 **	−0.27 **	228									
6. Cynicism(a)	3.60 (1.16)	0.55 **	0.66 **	−0.37 **	−0.51 **	0.64 **	228								
7. Vigor(a)	4.20 (1.05)	−0.29 **	−0.36 **	0.59 **	0.58 **	−0.29 **	−0.39 **	228							
8. Dedication(a)	4.31 (1.20)	−0.29 **	−0.46 **	0.47 **	0.63 **	−0.32 **	−0.53 **	0.79 **	228						
9. Organizational Deviance	1.70 (0.67)	0.23 **	0.24 **	−0.07	−0.13	0.11	0.15*	−0.06	−0.12	209					
10. Interpersonal Deviance	1.39 (0.52)	0.09	0.13	0.02	−0.02	0.04	0.18 **	0.01	−0.09	0.51 **	209				
11. OCB	4.26 (1.05)	−0.07	−0.09	0.32 **	0.32 **	−0.03	−0.11	0.23 **	0.16 *	−0.09	0.02	209			
12. JS	3.66 (1.02)	−0.29 **	−0.39 **	0.33 **	0.41 **	−0.27 **	−0.35 **	0.30 **	0.41 **	−0.28 **	−0.13	0.22 **	209		
13. OC	4.11 (1.21)	−0.29 **	−0.37 **	0.43 **	0.52 **	−0.30 **	−0.40 **	0.41 **	0.49 **	−0.21 **	−0.08	0.36 **	0.63 **	209	
14. SQ	4.04 (1.35)	−0.13	−0.13	0.26 **	0.24 **	−0.19 **	−0.16 *	0.20 **	0.15 *	−0.08	−0.005	0.24 **	0.23 **	0.18 **	209

Note. “f” indicates frequency response, while “a” indicates agreement response. Ns are shown on the diagonal. * *p* < 0.05. ** *p* < 0.01.

**Table 3:** Mean, standard deviation, and correlation matrix for Sample 2 (*N* = 281).

**Variable**	**M (SD**)	**1**	**2**	**3**	**4**	**5**	**6**	**7**	**8**	**9**	**10**	**11**	**12**	**13**	**14**
1. Exhaustion(f)	2.69 (1.27)	239													
2. Cynicism(f)	2.02 (1.20)	0.73 **	239												
3. Vigor(f)	5.82 (1.11)	−0.34 **	−0.41 **	239											
4. Dedication(f)	6.09 (1.01)	−0.31 **	−0.45 **	0.91 **	239										
5. Exhaustion(a)	2.91 (1.43)	0.24 **	0.24 **	−0.17 *	−0.19 **	258									
6. Cynicism(a)	2.23 (1.23)	0.23 **	0.20 **	−0.20 **	−0.20 **	0.65 **	258								
7. Vigor(a)	5.78 (0.94)	−0.15 *	−0.26 **	0.36 **	0.34 **	−0.44 **	−0.47 **	258							
8. Dedication(a)	6.17 (0.84)	−00.12	−0.18 **	0.35 **	0.33 **	−0.39 **	−0.51 **	0.84 **	258						
9. Physical fatigue(f)	2.08 (1.10)	0.66 **	0.65 **	−0.52 **	−0.51 **	0.20 **	0.13	−0.19 **	−0.15 *	239					
10. Cognitive weariness(f)	1.93 (1.09)	0.57 **	0.62 **	−0.49 **	−0.48 **	0.14 *	0.13	−0.20 **	−0.13	0.86 **	239				
11. Emotional exhaustion(f)	1.81 (1.12)	0.47 **	0.61 **	−0.43 **	−0.41 **	0.13	0.17 *	−0.20 **	−0.13	0.71 **	0.81 **	239			
12. Physical strength(f)	6.08 (1.17)	−0.51 **	−0.61 **	0.68 **	0.68 **	−0.22 **	−0.23 **	0.32 **	0.29 **	−0.62 **	−0.57 **	−0.52 **	239		
13. Cognitive liveliness(f)	5.73 (1.31)	−0.50 **	−0.55 **	0.71 **	0.68 **	−0.21 **	−0.19 **	0.30 **	0.25 **	−0.62 **	−0.63 **	−0.52 **	0.86 **	239	
14. Emotional energy(f)	5.94 (1.28)	−0.40 **	−0.50 **	0.63 **	0.60 **	−0.23 **	−0.22 **	0.32 **	0.25 **	−0.53 **	−0.52 **	−0.48 **	0.80 **	0.81 **	239
15. Physical fatigue(a)	2.19 (1.27)	0.22 **	0.26 **	−0.23 **	−0.22 **	0.70 **	0.74 **	−0.50 **	−0.47 **	0.21 **	0.16 *	0.16 *	−0.23 **	−0.19 **	−0.22 **
16. Cognitive weariness(a)	1.82 (1.08)	0.18 **	0.25 **	−0.23 **	−0.23 **	0.55 **	0.71 **	−0.55 **	−0.57 **	0.22 **	0.25 **	0.26 **	−0.23 **	−0.22 **	−0.23 **
17. Emotional exhaustion(a)	1.80 (1.08)	0.17 *	0.30 **	−0.24 **	−0.26 **	0.52 **	0.67 **	−0.58 **	−0.60 **	0.21 **	0.23 **	0.24 **	−0.19 **	−0.18 **	−0.19 **
18. Physical strength(a)	6.01 (1.02)	−0.21 **	−0.29 **	0.30 **	0.31 **	−0.56 **	−0.54 **	0.76 **	0.69 **	−0.19 **	−0.15*	−0.14 *	0.31 **	0.27 **	0.26 **
19. Cognitive liveliness(a)	6.09 (0.94)	−0.13 *	−0.26 **	0.35 **	0.35 **	−0.51 **	−0.54 **	0.75 **	0.73 **	−0.23 **	−0.25 **	−0.21 **	0.30 **	0.33 **	0.30 **
20. Emotional energy(a)	6.15 (0.93)	−0.15*	−0.26 **	0.37 **	0.35 **	−0.50 **	−0.55 **	0.74 **	0.74 **	−0.18 **	−0.16 *	−0.18 **	0.30 **	0.30 **	0.28 **
21.Organizational Deviance	1.44 (0.85)	0.16*	0.26 **	−0.24 **	−0.22 **	0.08	0.11	−0.15 *	−0.14	0.31 **	0.25 **	0.17 *	−0.29 **	−0.24 **	−0.22 **
22. Interpersonal Deviance	1.25 (0.76)	0.21 **	0.27 **	−0.29 **	−0.23 **	0.05	0.08	−0.19 **	−0.20 **	0.37 **	0.33 **	0.19 **	−0.32 **	−0.27 **	−0.22 **
23. OCB	5.37 (0.98)	−0.26 **	−0.27 **	0.30 **	0.27 **	0.03	−0.01	0.12	0.09	−0.25 **	−0.25 **	−0.24 **	0.31 **	0.34 **	0.30 **
24. JS	5.24 (0.96)	−0.24 **	−0.22 **	0.25 **	0.22 **	−0.05	−0.08	0.16*	0.14	−0.22 **	−0.17*	−0.15*	0.21 **	0.24 **	0.22 **
25. OC	6.14 (0.98)	−0.16 *	−0.28 **	0.22 **	0.23 **	−0.01	0.02	0.13	0.12	−0.25 **	−0.26 **	−0.17 *	0.24 **	0.26 **	0.24 **
26. SD	2.78 (1.25)	0.21 **	0.17 *	−0.15 *	−0.14	0.03	−0.03	−0.04	−0.06	0.24 **	0.16*	0.14	−0.13	−0.11	−0.17 *
27. WFS	5.77 (1.23)	−0.19 **	−0.23 **	0.19 *	0.202 **	0.05	0.002	0.10	0.05	−0.22 **	−0.19 **	−0.16 *	0.24 **	0.27 **	0.24 **
28. WFC	2.74 (1.26)	0.29 **	0.26 **	−0.18 *	−0.164 *	0.10	0.13	−0.14	−0.12	0.27 **	0.19 *	0.15 *	−0.18 *	−0.20 **	−0.19 **
**Variable**	**15**	**16**	**17**	**18**	**19**	**20**	**21**	**22**	**23**	**24**	**25**	**26**	**27**	**28**	
15. Physical fatigue(a)	258														
16. Cognitive weariness(a)	0.79 **	258													
17. Emotional exhaustion(a)	0.73 **	0.89 **	258												
18. Physical strength(a)	−0.68 **	−0.57 **	−0.53 **	258											
19. Cognitive liveliness(a)	−0.62 **	−0.69 **	−0.67 **	0.74 **	258										
20. Emotional energy(a)	−0.59 **	−0.66 **	−0.69 **	0.74 **	0.84 **	258									
21.Organizational Deviance	0.12	0.14	0.13	−0.17 *	−0.18 *	−0.24 **	188								
22. Interpersonal Deviance	0.10	0.16 *	0.14	−0.16 *	−0.21 **	−0.26 **	0.84 **	188							
23. OCB	0.03	−0.04	0.002	0.10	0.16 *	0.15 *	−0.24 **	−0.25 **	188						
24. JS	−0.07	−0.08	−0.06	0.15*	0.15 *	0.19 *	−0.18 *	−0.19 *	0.45 **	188					
25. OC	−0.04	−0.04	−0.09	0.09	0.16 *	0.17 *	−0.31 **	−0.29 **	0.40 **	0.49 **	188				
26. SD	0.04	0.01	0.07	0.05	−0.05	−0.11	0.33 **	0.34 **	−0.21 **	−0.38 **	−0.37 **	188			
27. WFS	0.04	−0.02	−0.01	0.06	0.14	0.13	−0.20 **	−0.23 **	0.57 **	0.51 **	0.51 **	−0.33 **	188		
28. WFC	0.17 *	0.14	0.10	−0.18*	−0.15*	−0.17*	0.35 **	0.26 **	−0.29 **	−0.54 **	−0.37 **	0.41 **	−0.33 **	188	

Note. Same as those in Table 2. * *p* < 0.05. ** *p* < 0.01.

**Table 4:** Relative importance analysis for Sample 1 (*N* = 242).

Variable	Raw Importance Estimates RW_j_	Rescaled Estimates	Incremental Importance (Δ*R*^2^)
DV = Organizational Deviance (Δ*R*^2^ = 0.07 *)			
1. Total-agreement	0.015	20%	0.01
2. Total-frequency	0.059	80%	0.05 *
DV = Interpersonal Deviance (Δ*R*^2^ = 0.07 †)			
1. Total-agreement	0.047	70%	0.04 †
2. Total-frequency	0.020	30%	0.01
DV = OCB (Δ*R*^2^ = 0.12 **)			
1. Total-agreement	0.026	22%	0.005
2. Total-frequency	0.095	78%	0.07 **
DV = General Job Satisfaction (Δ*R*^2^ = 0.26 ***)			
1. Total-agreement	0.119	47%	0.04 *
2. Total-frequency	0.149	53%	0.06 **
DV = Organizational Affective Commitment (Δ*R*^2^ = 0.33 ***)			
1. Total-agreement	0.161	48%	0.04 *
2. Total-frequency	0.172	52%	0.06 **
DV = Sleep quality (Δ*R*^2^ = 0.09 *)			
1. Total-agreement	0.037	42%	0.02
2. Total-frequency	0.050	58%	0.03

Note. Rescaled importance estimates were calculated by dividing the relative weights (RW_j_) by model R^2^. Because of rounding error, the values for RW_j_ may not sum to the model R^2^ and the values for rescaled estimates may not sum to unity [19]. The comparison measurement variables include vigor, dedication, exhaustion, and cynicism, with both frequency-based and agreement-based measurements. † *p* < 0.10; * *p* < 0.05; ** *p* < 0.01; *** *p* < 0.001.

**Table 5:** Relative importance analysis for Sample 2 Surveys I and II (*N* = 281).

Variable	Raw Importance Estimates RW_j_		Rescaled Estimates		Incremental Importance (Δ*R*^2^)	
DV = Organizational Deviance (Δ*R*^2^ = 0.10 *)						
	I	II	I	II	I	II
1. Total-agreement	0.011	0.041	11%	26%	0.003	0.03
2. Total-frequency	0.090	0.115	89%	74%	0.08 **	0.10 **
DV = Interpersonal Deviance (Δ*R*^2^ = 0.13 **)						
	I	II	I	II	I	II
1. Total-agreement	0.028	0.051	21%	22%	0.01	0.04
2. Total-frequency	0.106	0.181	79%	78%	0.09 **	0.16 ***
DV = OCB (Δ*R*^2^ = 0.140 ***)						
	I	II	I	II	I	II
1. Total-agreement	0.018	0.050	13%	31%	0.02	0.04
2. Total-frequency	0.124	0.111	87%	69%	0.12 ***	0.09 **
DV = General Job Satisfaction (Δ*R*^2^ = 0.08 †)						
	I	II	I	II	I	II
1. Total-agreement	0.018	0.028	21%	36%	0.01	0.02
2. Total-frequency	0.066	0.051	79%	64%	0.06 *	0.04
DV = Organizational Affective Commitment (Δ*R*^2^ = 0.11 **)						
	I	II	I	II	I	II
1. Total-agreement	0.015	0.048	14%	38%	0.01	0.04
2. Total-frequency	0.095	0.081	86%	62%	0.09 **	0.07 *
DV = Sleep disorder (Δ*R*^2^ = 0.06)						
	I	II	I	II	I	II
1. Total-agreement	0.010	0.087	16%	52%	0.01	0.08 *
2. Total-frequency	0.053	0.080	84%	58%	0.05 *	0.07 *
DV = Work-family positive spillover (Δ*R*^2^ = 0.09 *)						
	I	II	I	II	I	II
1. Total-agreement	0.021	0.045	24%	40%	0.02	0.04
2. Total-frequency	0.067	0.066	76%	60%	0.06 *	0.05
DV = Work-family conflict (Δ*R*^2^ = 0.10 *)						
	I	II	I	II	I	II
1. Total-agreement	0.015	0.036	14%	31%	0.01	0.02
2. Total-frequency	0.088	0.077	86%	69%	0.08 **	0.07 *

Note. Same as those in Table 4. † *p* < 0.10; * *p* < 0.05; ** *p* < 0.01; *** *p* < 0.001.

## References

[1] Li P., Taris T.W., Peeters M.C.W. (2019). Challenge and hindrance appraisals of job demands: One man’s meat, another man’s poison?. Anxiety Stress Coping.

[2] Travis D.J., Lizano E.L., Mor Barak M.E. (2016). “I’m so stressed!”: A longitudinal model of stress, burnout and engagement among social workers in child welfare settings. Br. J. Soc. Work.

[3] Salmela-Aro K. (2017). Dark and bright sides of thriving—School burnout and engagement in the Finnish context. Eur. J. Dev. Psychol..

[4] Schaufeli W.B., Bakker A.B. (2003). UWES-Utrecht Work Engagement Scale: Test Manual.

[5] Saucier G. (1994). Mini-markers: A brief version of Goldberg’s unipolar big-five markers. J. Personal. Assess..

[6] Luthans F., Youssef C.M., Avolio B.J. (2007). Psychological Capital: Developing the Human Competitive Edge.

[7] Dunford B.B., Shipp A.J., Boss R.W., Angermeier I., Boss A.D. (2012). Is burnout static or dynamic? A career transition perspective of employee burnout trajectories. J. Appl. Psychol..

[8] Kühnel J., Sonnentag S., Bledow R. (2012). Resources and time pressure as day-level antecedents of work engagement. J. Occup. Organ. Psychol..

[9] Maslach C., Schaufeli W.B., Leiter M.P. (2001). Job burnout. Annu. Rev. Psychol..

[10] Newton D.W., LePine J.A., Kim J.K., Wellman N., Bush J.T. (2020). Taking engagement to task: The nature and functioning of task engagement across transitions. J. Appl. Psychol..

[11] Leon M.R., Halbesleben J.R.B., Paustian-Underdahl S.C. (2015). A dialectical perspective on burnout and engagement. Burn. Res..

[12] Marfeo E.E., Ni P., Chan L., Rasch E.K., Jette A.M. (2014). Combining agreement and frequency rating scales to optimize psychometrics in measuring behavioral health functioning. J. Clin. Epidemiol..

[13] Brown G.T. (2004). Measuring attitude with positively packed self-report ratings: Comparison of agreement and frequency scales. Psychol. Rep..

[14] Schwarz N. (1999). Self-reports: How the questions shape the answers. Am. Psychol..

[15] Bradburn N., Rips L., Shevell S. (1987). Answering autobiographical questions: The impact of memory and inference on surveys. Science.

[16] Sudman S., Bradburn N.M., Schwarz N. (1996). Thinking about Answers: The Application of Cognitive Processes to Survey Methodology.

[17] Leiter M.P., Maslach C. (2017). Burnout and engagement: Contributions to a new vision. Burn. Res..

[18] Tonidandel S., LeBreton J.M. (2011). Relative importance analysis: A useful supplement to regression analysis. J. Bus. Psychol..

[19] LeBreton J.M., Hargis M.B., Griepentrog B., Oswald F.L., Ployhart R.E. (2007). A multidimensional approach for evaluating variables in organizational research and practice. Pers. Psychol..

[20] Cooper W.H., Richardson A.J. (1986). Unfair comparisons. J. Appl. Psychol..

[21] Podsakoff P.M., MacKenzie S.B., Lee J.Y., Podsakoff N.P. (2003). Common method biases in behavioral research: A critical review of the literature and recommended remedies. J. Appl. Psychol..

[22] Maslach C., Jackson S.E., Leiter M.P. (1996). Maslach Burnout Inventory Manual.

[23] Demerouti E., Mostert K., Bakker A.B. (2010). Burnout and work engagement: A thorough investigation of the independency of both constructs. J. Occup. Health Psychol..

[24] Schaufeli W.B., Salanova M., González-romá V., Bakker A.B. (2002). The measurement of engagement and burnout: A confirmative analytic approach. J. Happiness Stud..

[25] Shirom A., Melamed S. (2006). A comparison of the construct validity of two burnout measures in two groups of professionals. Int. J. Stress Manag..

[26] Shirom A. (2003). Feeling vigorous at work? The construct of vigor and the study of positive affect in organizations. Res. Organ. Stress Well Being.

[27] Vallieres A., Azaiez A., Moreau V., LeBlanc M., Morin C.M. (2014). Insomnia in shift work. Sleep Med..

[28] Alarcon G.M. (2011). A meta-analysis of burnout with job demands, resources, and attitudes. J. Vocat. Behav..

[29] Christian M.S., Garza A.S., Slaughter J.E. (2011). Work engagement: A quantitative review and test of its relations with task and contextual performance. Pers. Psychol..

[30] Cole M.S., Walter F., Bedeian A.G., O’Boyle E.H. (2011). Job burnout and employee engagement. J. Manag..

[31] Swider B.W., Zimmerman R.D. (2010). Born to burnout: A meta-analytic path model of personality, job burnout, and work outcomes. J. Vocat. Behav..

[32] Van Jaarsveld D.D., Walker D.D., Skarlicki D.P. (2010). The role of job demands and emotional exhaustion in the relationship between customer and employee incivility. J. Manag..

[33] Wagner D.T., Barnes C.M., Scott B.A. (2014). Driving it home: How workplace emotional labor harms employee home life. Pers. Psychol..

[34] Hanson G.C., Hammer L.B., Colton C.L. (2006). Development and validation of a multidimensional scale of perceived work-family positive spillover. J. Occup. Health Psychol..

[35] Fox S., Spector P.E. (1999). A model of work frustration-aggression. J. Organ. Behav..

[36] Smith C.A., Organ D.W., Near J.P. (1983). Organizational citizenship behavior: Its nature and antecedents. J. Appl. Psychol..

[37] Podsakoff P.M., Ahearne M., MacKenzie S.B. (1997). Organizational citizenship behavior and the quantity and quality of work group performance. J. Appl. Psychol..

[38] Farh J.-L., Zhong C.-B., Organ D.W. (2004). Organizational citizenship behavior in the People’s Republic of China. Organ. Sci..

[39] Evers A., Frese M., Cooper C.L. (2000). Revisions and further developments of the Occupational Stress Indicator: LISREL results from four Dutch studies. J. Occup. Organ. Psychol..

[40] Chen Z.X., Francesco A.M. (2003). The relationship between the three components of commitment and employee performance in China. J. Vocat. Behav..

[41] American Psychiatric Association (2013). Diagnostic and Statistical Manual of Mental Disorders: DSM-5.

[42] Buysse D.J., Reynolds C.F., Monk T.H., Berman S.R., Kupfer D.J. (1989). The Pittsburgh Sleep Quality Index: A new instrument for psychiatric practice and research. Psychiatry Res..

[43] Kecklund G., Akerstedt T. (1992). The psychometric properties of the Karolinska Sleep Questionnaire. J. Sleep Res..

[44] Carlson D.S., Kacmar K.M., Williams L.J. (2000). Construction and initial validation of a multidimensional measure of work–family conflict. J. Vocat. Behav..

[45] Tong J., Wang L. (2012). Work locus of control and its relationship to stress perception, related affections, attitudes and behaviours from a domain-specific perspective. Stress Health.

[46] Fox S., Spector P.E., Goh A., Bruursema K., Kessler S.R. (2012). The deviant citizen: Measuring potential positive relations between counterproductive work behaviour and organizational citizenship behaviour. J. Occup. Organ. Psychol..

